# Context-Aware Online Model Splitting and Device Association for Semi-Decentralized Federated Learning in Internet of Things

**DOI:** 10.3390/s26134016

**Published:** 2026-06-24

**Authors:** Bo Xu, Shuang Wang, Xiaoyu Tang

**Affiliations:** 1 Jiangsu Key Laboratory of Wireless Communications, Nanjing University of Posts and Telecommunications, Nanjing 210003, China; 1025010240@njupt.edu.cn; 2 Zhengzhou Power Supply Company, State Grid Henan Electric Power Company, Zhengzhou 450015, China; wangshuang8@ha.sgcc.com.cn

**Keywords:** federated learning, split learning, latency optimization, multi-armed bandit

## Abstract

As a distributed approach to Artificial Intelligence (AI) model construction over wireless networks, federated learning (FL) based on multi-device collaborative training can protect data privacy, as well as increase the computing load of local model updates. In contrast, split learning (SL) with proper model splitting can adapt to the computation and transmission capabilities among devices. In this paper, while taking advantage of FL and SL, we concentrate on a semi-decentralized hybrid federated split learning (SD-HFSL) framework, in which we surpass the limitations of a single central server and allow the shared split models to be aggregated among multiple edge servers. To verify the importance of latency optimization for training efficiency, we analyze the convergence performance of SD-HFSL while jointly considering the limited computation and communication resources. Then, aiming at maximizing the long-term training efficiency, we propose an online optimization problem that includes local model splitting and device association. Considering that the training latency is unknown to the system a priori, a context-aware online training algorithm with sublinear regret is proposed based on the framework of contextual multi-armed bandit (CMAB), where the edge servers can observe the context information of device sites for latency estimation, followed by the iterative optimization based on the evaluated information in different contexts. Experiments on several neural network models show that the proposed algorithm reduces training latency and improves test accuracy compared with the selected benchmarks.

## 1. Introduction

Internet of Things (IoT) technology has been extensively applied across a variety of domains, including industry and transportation, where demand for intelligent applications continues to grow [1,2]. These intelligent applications are often implemented based on Artificial Intelligence (AI) technologies with a large amount of training data collected from widely distributed IoT devices [3]. Meanwhile, considering the increasing complexity of neural network models, it is more difficult for resource-constrained devices to efficiently build training models to support rapidly iterating AI applications. In particular, although cloud computing [4] or edge computing [5] has the ability to provide extra computing resources for large-scale data training, the third-party computing model has difficulty with meeting the privacy requirements among the involved devices [6]. To solve this issue, taking advantage of the distributed devices and their local computing power, the federated learning (FL) [7] algorithm is an approach that can be used for cooperatively training AI models by using the local data of several devices while meeting privacy requirements. In contrast to conventional centralized training, which exchanges local data with a central server to train on massive volumes of raw data, FL can effectively safeguard data privacy without local data interaction. Recent works have designed a variety of FL frameworks to accommodate a large number of devices by using techniques to reduce training overhead [8,9]. Moreover, to facilitate FL over dynamic wireless networks, strategies of resource allocation were fully addressed, considering communication link unreliability [2,10,11,12] and training efficiency [13,14]. Moreover, some interesting reviews of FL can be found in [15,16,17,18].

Nevertheless, with the increasing complexity of AI models, low-performance devices under the short-plate effect can significantly increase training latency and weaken the training efficiency of FL in real-world scenarios. Most importantly, the local computing tasks of traditional FL will be completely dependent on the device itself, which can not fully mobilize the existing computing resources. Considering the problem that a single device is insufficient to support complex training tasks, a deep neural network (DNN) can be divided into several sections via split learning (SL) [19], which is a cooperative training method that can send procedures of forward propagation and gradient calculations to other computing nodes for processing, such as devices and edge servers. Compared with FL, SL can lower a single device’s processing load and energy usage by performing model splitting and distributing the intermediate outcomes, such as model features and gradients across other computing units. To reduce the computational load of model training and inference processes, the SL framework is implemented in open-source applications, such as indoor positioning, cooperative identification, and rapid reasoning [20]. Moreover, in academia, SL is deployed over wireless networks, maximizing learning performance with resource adaptation [21,22,23,24].

However, the amount of data possessed by a single device in SL is limited, and the trained model is insufficient in generalization ability or learning ability, especially in the heterogeneous data environment. To solve this issue, jointly considering the advantages of FL and SL, recent works have incorporated the low computational load on SL devices and collaborative training of FL [25,26,27,28,29,30,31,32,33]. In [25], a novel learning framework, namely, hybrid federated split learning (HSFL), was proposed to obtain a trade-off between training time and energy consumption with the joint optimization problem, including splitting decisions. Moreover, the time consumption of the stragglers in HSFL was considered in [26], where the local models were trained among all devices along the ring through a pre-defined direction. We can see that HSFL, in fact, retains the characteristics of FL, integrated with existing learning mechanisms. For instance, in [27], a special split from the perspective of clustering was proposed in HFSL, which was similar to clustered FL, but optimized the model updating and cluster splitting schemes in the training stage to accelerate model convergence. Moreover, the authors in [28] adapted HFSL to intrusion detection systems, considering the reliability of split information in the sharing process. To strike a balance between latency and performance, optimization was proposed in [29] by jointly optimizing client selection, model splitting, and bandwidth allocation policies. Recent work [30] analyzed and optimized the HFSL under multi-tier systems, improving the flexibility of model splitting. An asynchronous framework that enabled personalized model splitting and aperiodic model aggregation was proposed in [31] to minimize the long-term average training latency. Moreover, HFSL can also introduce differential privacy and other security mechanisms under the premise of ensuring learning efficiency [32,33]. These studies indicate that HFSL can combine the low local computational load of SL with the collaborative training ability of FL.

With regard to HFSL, there is a practical issue that has not been fully considered in previous works. In particular, the current HFSL framework is trained under the presumption that a central server has complete control over learning, which is obviously difficult to achieve due to the fact that the communication and computing power of the central server are limited. In particular, when the hardware of the central server fails, the local model of the device will lose the object of model uploading, resulting in extremely severe stagnation of the training process. To solve this issue, semi-decentralized edge learning frameworks were recently proposed in [34,35,36], which can greatly improve the robustness of the training process by performing model aggregation among various edge servers without a central server. Specifically, to reduce the consumption of spectrum resources, an adaptive control technique combining edge aggregation with device-to-device (D2D) technologies was proposed in [34]. Moreover, under the multiple edge server scenario, a tradeoff between training effectiveness and energy consumption was considered in [35]. Additionally, convergence performance of the global model in the case of decentralized architecture was analyzed in [36]. HFSL can be integrated with a semi-central architecture by reorganizing the local model update process into the interaction process between devices and edge servers. However, this combination is not simple to achieve and will create new challenges. On the one hand, adding the model splitting mechanism under a decentralized architecture needs to be redesigned according to the characteristics of each device in order to improve the training efficiency with limited wireless resources. On the other hand, it is difficult to describe the convergence performance of HFSL in a scenario of multiple edge servers accurately, and there is a lack of correspondence between training efficiency and splitting strategy. Therefore, we need to design an efficient training mechanism that includes model splitting and device association, where a semi-decentralized hybrid federated split learning (SD-HFSL) framework is designed.

In this paper, the considered SD-HFSL framework can use SL to reduce the computational load of FL devices with intra- and inter-aggregations among multiple edge servers. Aiming at maximizing the long-term training efficiency, an online problem of model splitting decisions is formulated. Specifically, considering that the training latency is uncertain in different states of devices, i.e., the communication environment varies across time, and the local computing power changes accordingly. Learning the training latency for each device site precisely with a cold start (i.e., no prior knowledge available) is the first step toward efficient model splitting. Fortunately, to solve this issue, multi-armed bandit (MAB) algorithms have been studied to address the tradeoff between exploration and exploitation in sequential decision-making with uncertain information. The classic MAB algorithm, e.g., upper confidence bound (UCB), is concerned with learning the single optimal action among a set of candidate actions with unknown rewards [37]. Using the known information adequately, contextual bandits extend the basic MAB to contextual multi-armed bandit (CMAB) [38,39], where the edge servers can observe the context information (i.e., channel state, allocated bandwidth, transmit power, interference) of device sites for the training latency estimation, especially local computing power estimation. Since the problem of contextual bandits is already much more common and difficult than the basic MAB, this paper tackles solutions based on the framework of CMAB. Different from the previous works [38,39], this paper can merge local computing power evaluation with the training process by considering the training, exploration, and exploitation phases jointly. Meanwhile, we investigate multiple servers with incomplete information and compare them with the existing works of HFSL. Finally, the considered SD-HFSL framework based on the proposed scheme can improve learning efficiency, even with limited resources and unknown information. As summarized in Table 1, the main technical distinction of this work is not merely combining FL and SL, but jointly considering three coupled aspects that are usually treated separately, including semi-decentralized aggregation among multiple edge servers, three-part split training without label uploading, and context-aware online optimization of both split points and device–server association under unknown latency.

As such, the main contributions of this paper are as follows.

- We propose a novel SD-HFSL framework over wireless networks, where multiple edge servers are deployed, and each of them can coordinate a device cluster with splitting-based local model updating. Moreover, the edge models can be periodically updated based on intra-cluster model aggregation and inter-cluster model aggregation. According to the analysis results of convergence performance, aiming at maximizing the long-term training efficiency, an online optimization problem of model splitting and device association is formulated.
- An online decision-making algorithm based on the framework of CMAB is proposed, which allows the edge servers to observe the context information of device sites for training latency estimation. Meanwhile, combined with the evaluated information, devices can update the model splitting and association decisions according to the estimated context-dependent latency through exploration and exploitation in sequential decision-making under uncertainty. Meanwhile, we prove that the proposed algorithm can provide a provable performance, achieving sublinear regret compared to an oracle algorithm that knows the expected training latency.
- Our experiments adapt several model structures, including AlexNet, VGG16, and ResNet18, and present several comparison algorithms based on the existing works. The simulation results show that the proposed algorithm achieves lower training latency and higher test accuracy in the considered settings, especially when prior latency information is unavailable.

The organization of this paper is structured as follows. The system model with convergence analysis is presented in Section 2. The long-term optimization problem is introduced in Section 3. The details of the proposed online algorithm for training efficiency improvement are introduced in Section 4. Finally, we present extensive experimental results in Section 5, and the conclusions are drawn in Section 6.

## 2. System Model

### 2.1. SD-HFSL Framework

**Figure 1:**
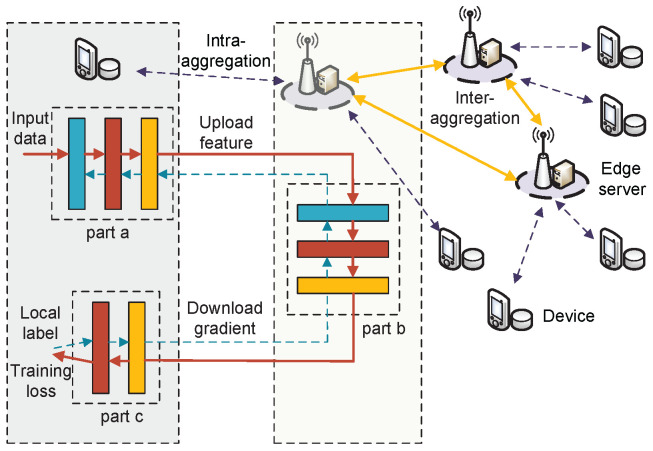
The considered SD-HFSL framework over wireless networks.

The proposed SD-HFSL framework is shown in Figure 1, which consists of a set of edge servers S={1,2,…,S} and a set of devices K={1,2,…,K}.

Each device k∈K has its local dataset ${\left\{x_{k,i},y_{k,i}\right\}}_{i=1}^{D_{k}}$ with $D_{k}$ training samples, where $x_{k,i}$ and $y_{k,i}$ are the *i*-th training data and the corresponding training label, respectively. Given the training round set T={1,2,…,T}, by performing *T* rounds of multi-devices collaborative learning, our goal is to find the optimal global model ${\bar{w}}^{*}\left(T\right)$ from the trained edge model set $\bar{W}\left(T\right)=\left\{{\bar{w}}_{1}\left(T\right),{\bar{w}}_{2}\left(T\right),\ldots ,{\bar{w}}_{S}\left(T\right)\right\}$ that can minimize the global training loss, i.e.,

(1) $$\min_{\bar{w}\left(T\right)\in \bar{W}\left(T\right)}F\left(\bar{w}\left(T\right)\right)=\frac{\sum_{k\in K}\left|D_{k}\right|f_{k}\left(\bar{w}\left(T\right)\right)}{\sum_{k\in K}\left|D_{k}\right|},$$

where $f_{k}\left(\bar{w}\left(T\right)\right)$ is the local loss of device *k* with edge model $\bar{w}\left(T\right)$ and can be evaluated at the end of each training round.

The main notation used in the system model is summarized in Table 2.

Specially, compared with the training process in traditional semi-decentralized FL [40], the characteristic of the considered SD-HFSL framework is that we redesign the device and edge server model interaction process, assuming that the trained local model on the device can be split to offload part of the computation to the associated edge server. For instance, we design a three-part splitting strategy without label sharing [26], where the DNN is beyond the execution capacity of the device and can be split into three parts, namely part *a*, part *b*, and part *c*. It is divided into three parts because we consider that in the case of model splitting, the labels of devices do not leave the local area in the gradient descent calculation, so as to improve the privacy protection capabilities. We note that keeping labels locally does not eliminate all privacy risks because intermediate features and back-propagated gradients may still contain information about local samples. Therefore, the proposed framework focuses on reducing label exposure and communication/computation latency, while stronger protection can be incorporated by adding feature perturbation, differential privacy noise, or secure aggregation to the exchanged activations and gradients.

Then, in each training round *t*, the key steps of the SD-HFSL framework include local model updates, intra-cluster model aggregation, and inter-cluster model aggregation.

(1) *Local Model Updates*: Considering the case of model splitting, the initial parameter w(t) of the device *k* in training round *t* can be written as $w_{k}\left(t\right)=\left\{w_{k}^{a}\left(t\right),w_{k}^{b}\left(t\right),w_{k}^{c}\left(t\right)\right\}$, where only the model parameters of part *a* and part *c* are downloaded to devices. Since the model in splitting is usually large in scale and occupies more computing resources, we adopt parallel SGD with fast aggregation, in which the local model $w_{k}\left(t\right)$ obtained by device *k* is updated as

(2) $$w_{k}\left(t\right)=w_{k}\left(t-1\right)-\eta g_{k}\left(\zeta_{k}\left(t\right),w_{k}\left(t-1\right)\right),$$

where $g(\zeta_{k}\left(t\right),w_{k}\left(t-1\right))$ is the gradient computed on the batch of randomly-sampled local training dataset $\zeta_{k}\left(t\right)$ with model parameter $w_{k}\left(t-1\right)$, and η is the learning rate. Moreover, the parameter update for devices with splitting is performed based on the vanilla SL without label sharing. Specifically, in the forward propagation of SL, $w_{k}^{a}\left(t\right)$ is executed locally and the output feature is uploaded to the associated edge servers for the calculation of $w_{k}^{b}\left(t\right)$. Then, the output of the edge server is downloaded by the device for the execution of $w_{k}^{c}\left(t\right)$. Similarly, in the backward propagation, the gradients of part *c*, part *b* and part *a* are calculated in order, denoted as $g_{k}^{c}\left(w_{k}\left(t\right)\right)$, $g_{k}^{b}\left(w_{k}\left(t\right)\right)$, and $g_{k}^{a}\left(w_{k}\left(t\right)\right)$, respectively.

(2) *Intra-cluster Model Aggregation*: When the local models are updated, each device can upload its complete or partial model parameters to the associated edge server, performing intra-cluster model aggregation to obtain the edge mode ${\tilde{w}}_{s}\left(t\right)$, i.e.,

(3) $${\tilde{w}}_{s}\left(t\right)=\frac{\sum_{k\in V_{s}\left(t\right)}D_{k}w_{k}\left(t\right)}{\sum_{k\in V_{s}\left(t\right)}D_{k}},$$

where the set of devices associated with the *s*-th edge server and successfully participating in training round *t* is denoted as $V_{s}\left(t\right)$. Then, the updated edge models can be broadcast to the associated devices for the following local model updates in the current training round.

(3) *Inter-cluster Model Aggregation*: When the edge model is updated, each edge server can upload its model parameters to the associated edge servers, performing inter-cluster model aggregation, i.e.,

(4) $$\begin{array}{cc} \; & {\tilde{w}}_{s}\left(t+1\right)=\\ & \frac{\sum_{k\in V_{s}\left(t\right)}D_{k}w_{k}\left(t\right)+\sum_{s^{\prime }\in C_{s}\left(t\right)}\sum_{k^{\prime }\in V_{s^{\prime }}\left(t\right)}D_{k^{\prime }}w_{k^{\prime }}\left(t\right)}{\sum_{k\in V_{s}\left(t\right)}D_{k}+\sum_{s^{\prime }\in C_{s}\left(t\right)}\sum_{k^{\prime }\in V_{s^{\prime }}\left(t\right)}D_{k^{\prime }}}, \end{array}$$

where $C_{s}\left(t\right)$ is the set of edge servers that are placed in the converging area of edge server *s* with successful model interaction in training round *t*.

Finally, at the end of each training round, the updated edge models of edge servers are transmitted to the associated devices to perform the next round of training. As the complexity of the model increases, it is usually required to perform many training rounds to obtain the desired learning performance.

### 2.2. Latency Model

The latency of the SD-HFSL framework consists of forward propagation and back propagation. Meanwhile, the single-round training process involves multiple stages of model transmission and computation.

(1) *Transmission Latency*: The number of divisible layers contained in a model is denoted as $I^{\max}$. Then, let $1\leq I_{k}^{a,b}\left(t\right)\leq I^{\max}-2$ and $I_{k}^{a,b}\left(t\right)<I_{k}^{b,c}\left(t\right)\leq I^{\max}-1$ be the two breakpoints of the breakpoints for device *k* in training round *t*, corresponding to the model, divided into three parts. In addition, if the local model of device *k* is not split, we can derive that $I_{k}^{a,b}\left(t\right)=I_{k}^{b,c}\left(t\right)=0$. To avoid ambiguity between association and successful participation, we use $x_{k,s}\left(t\right)$ to denote the device–server association before training latency is evaluated. After $\tau_{k}\left(t\right)$ is obtained, $z_{k}\left(t\right)$ denotes whether device *k* satisfies the latency budget, and $a_{k,s}\left(t\right)=x_{k,s}\left(t\right)z_{k}\left(t\right)$ denotes effective participation in aggregation and utility evaluation.

In the forward propagation of device *k*, denote $T_{k}^{U}\left(t\right)=\left\{\tau_{k}^{U,a,b}\left(t\right),\tau_{k}^{U,b,c}\left(t\right)\right\}$ as the uploading latency set, where $\tau_{k}^{U,a,b}\left(t\right)$ and $\tau_{k}^{U,b,c}\left(t\right)$ are the latencies of uploading the output features of part *a* to part *b*, and part *b* to part *c*, respectively. Mathematically, we can evaluate $\tau_{k}^{U,a,b}\left(t\right)$ and $\tau_{k}^{U,b,c}\left(t\right)$ with feature size $M_{k}^{U,a,b}\left(t\right)$ and $M_{k}^{U,b,c}\left(t\right)$ as

(5) $$\begin{array}{cc} \tau_{k}^{U,a,b}\left(t\right) & =I\left(I_{k}^{a,b}\left(t\right)\right)\sum_{s\in S}\frac{x_{k,s}\left(t\right)M_{k}^{U,a,b}\left(t\right)}{W_{k,s}^{a,b}\left(t\right)\log_{2}\left(1+\frac{p_{k}\left(t\right)H_{k,s}\left(t\right)}{n_{k}^{\mathrm{intra-cluster}}\left(t\right)+n_{k}^{\mathrm{inter-cluster}}\left(t\right)+\sigma^{2}}\right)}\\ & +\left(1-I\left(I_{k}^{a,b}\left(t\right)\right)\right)\tau_{k}^{U,0}\left(t\right) \end{array}$$

and

(6) $$\tau_{k}^{U,b,c}\left(t\right)=I\left(I_{k}^{b,c}\left(t\right)\right)\sum_{s\in S}\frac{x_{k,s}\left(t\right)M_{k}^{U,b,c}\left(t\right)}{{\tilde{W}}_{s,k}^{b,c}\left(t\right)\log_{2}\left(1+\frac{{\tilde{p}}_{s,k}\left(t\right){\tilde{H}}_{s,k}\left(t\right)}{{\tilde{n}}_{s}^{\mathrm{intra-cluster}}\left(t\right)+{\tilde{n}}_{s}^{\mathrm{inter-cluster}}\left(t\right)+\sigma^{2}}\right)},$$

respectively, where I(·) is the indicator function that determines whether there is model splitting, $\tau_{k}^{U,0}\left(t\right)$ is the latency for forward propagation without considering model splitting, $W_{k,s}^{a,b}\left(t\right)$ is the bandwidth allocated to the device *k*, $p_{k}\left(t\right)$ refers to the transmit power of device *k*, $H_{k,s}\left(t\right)$ is the channel gain between the device *k* and its associated edge server, $n_{k}^{\mathrm{intra-cluster}}\left(t\right)$ is the intra-cluster interference caused by the devices associated with the same edge server, $n_{k}^{\mathrm{inter-cluster}}\left(t\right)$ is the inter-cluster interference caused by the other elements in the system with the same frequency, and $\sigma^{2}$ is the additive white Gaussian noise (AWGN). Similarly, ${\tilde{W}}_{s,k}^{b,c}\left(t\right)$ is the bandwidth allocated to the edge server to transmit model parameters to device *k*, ${\tilde{p}}_{s,k}\left(t\right)$ refers to the transmit power of edge server *s*, ${\tilde{H}}_{s,k}\left(t\right)$ is the channel gain between the device *k* and its associated edge server *s*, and ${\tilde{n}}_{s}^{\mathrm{intra-cluster}}\left(t\right)$ and ${\tilde{n}}_{s}^{\mathrm{inter-cluster}}\left(t\right)$ are the intra- and inter-cluster interferences for edge server *s*. Similarly, the transmission latency in back propagation is denoted as $T_{k}^{D}\left(t\right)=\left\{\tau_{k}^{D,c,b}\left(t\right),\tau_{k}^{D,b,a}\left(t\right)\right\}$. Assuming the channel and noise environment of the system in the process of feature uploading and gradient downloading are unchanged in the same training round, let ${\bar{M}}_{k}^{D,c,b}\left(t\right)$ and ${\bar{M}}_{k}^{D,b,a}\left(t\right)$ be the size of the gradients determined by the split points for device *k* in training round *t*, then we can evaluate $\tau_{k}^{D,c,b}\left(t\right)$ and $\tau_{k}^{D,b,a}\left(t\right)$ as

(7) $$\begin{array}{cc} \tau_{k}^{D,c,b}\left(t\right) & =I\left(I_{k}^{b,c}\left(t\right)\right)\frac{{\bar{M}}_{k}^{D,c,b}\left(t\right)}{M_{k}^{U,b,c}\left(t\right)}\tau_{k}^{U,b,c}\left(t\right) \end{array}$$

and

(8) $$\begin{array}{c} \tau_{k}^{D,b,a}\left(t\right)=I\left(I_{k}^{a,b}\left(t\right)\right)\frac{{\bar{M}}_{k}^{D,b,a}\left(t\right)}{M_{k}^{U,a,b}\left(t\right)}\tau_{k}^{U,a,b}\left(t\right), \end{array}$$

respectively.

(2) *Computing Latency*: Denote $C^{F,l}$ and $C^{B,l}$ as the number of floating point operations (FLOPs) required by the *l*-th layer in the forward and backward propagation. Moreover, the local computing power in cycle/s of device *k* and edge server *s* are denoted as $f_{k}\left(t\right)$ and ${\tilde{f}}_{s}\left(t\right)$, respectively. Then, the computing latency neural networks in forward propagation for part *a*, part *b*, and part *c* can be evaluated as

(9) $$\begin{array}{cc} \tau_{k}^{C,a}\left(t\right) & =I\left(I_{k}^{a,b}\left(t\right)\right)\sum_{l=1}^{I_{k}^{a,b}\left(t\right)}\frac{C^{F,l}}{f_{k}\left(t\right)}+\left(1-I\left(I_{k}^{a,b}\left(t\right)\right)\right)\tau_{k}^{C,0}\left(t\right), \end{array}$$

(10) $$\tau_{k}^{C,b}\left(t\right)=I\left(I_{k}^{b,c}\left(t\right)\right)\sum_{s\in S}x_{k,s}\left(t\right)\sum_{l=I_{k}^{a,b}\left(t\right)+1}^{I_{k}^{b,c}\left(t\right)}\frac{C^{F,l}}{{\tilde{f}}_{s}\left(t\right)},$$

and

(11) $$\tau_{k}^{C,c}\left(t\right)=I\left(I_{k}^{b,c}\left(t\right)\right)\sum_{l=I_{k}^{b,c}\left(t\right)+1}^{I^{\max}}\frac{C^{F,l}}{f_{k}\left(t\right)},$$

respectively, where $\tau_{k}^{C,0}\left(t\right)$ can be the computing latency for forward propagation without considering model splitting in device *k*. Similarly, let $\tau_{k}^{B,0}\left(t\right)$ be the computing latency for back propagation without considering model splitting in device *k*. Then, the computing latency in back propagation can be evaluated as

(12) $$\begin{array}{c} \tau_{k}^{G,c}\left(t\right)=I\left(I_{k}^{b,c}\left(t\right)\right)\sum_{l=I_{k}^{b,c}\left(t\right)+1}^{I^{\max}}\frac{C^{B,l}}{f_{k}\left(t\right)}+\left(1-I\left(I_{k}^{a,b}\left(t\right)\right)\right)\tau_{k}^{B,0}\left(t\right), \end{array}$$

(13) $$\tau_{k}^{G,b}\left(t\right)=I\left(I_{k}^{b,c}\left(t\right)\right)\sum_{s\in S}x_{k,s}\left(t\right)\sum_{l=I_{k}^{a,b}\left(t\right)+1}^{I_{k}^{b,c}\left(t\right)}\frac{C^{B,l}}{{\tilde{f}}_{s}\left(t\right)},$$

and

(14) $$\tau_{k}^{G,a}\left(t\right)=I\left(I_{k}^{a,b}\left(t\right)\right)\sum_{l=1}^{I_{k}^{a,b}\left(t\right)}\frac{C^{B,l}}{f_{k}\left(t\right)},$$

respectively.

In addition, similar to traditional FL, the SD-HFSL framework can also be designed for synchronous aggregation. The dominant latency terms are the split forward/backward computation and feature/gradient transmission. The aggregation, inter-edge exchange, model broadcast, and edge queuing delays are therefore omitted in the main expression for analytical tractability. If these components are non-negligible in a practical deployment, they can be added as an extra term $\tau^{\mathrm{agg}}\left(t\right)+\tau^{\mathrm{inter}}\left(t\right)+\tau^{\mathrm{bc}}\left(t\right)+\tau^{q}\left(t\right)$ without changing the proposed online decision structure, where $\tau^{\mathrm{agg}}\left(t\right)$ denotes the intra-cluster aggregation latency, $\tau^{\mathrm{inter}}\left(t\right)$ denotes the inter-edge model exchange latency, $\tau^{\mathrm{bc}}\left(t\right)$ denotes the model broadcast latency from edge servers to devices, and $\tau^{q}\left(t\right)$ denotes the edge-server queuing latency in training round *t*.

Thus, without considering the latency of model aggregation, inter-cluster model interaction, and model broadcast, we can evaluate the actual training latency of the system in round *t* as

(15) $$\begin{array}{cc} \tau (t) & =\max_{k\in K}\tau_{k}\left(t\right), \end{array}$$

where

(16) $$\begin{array}{cc} \tau_{k}\left(t\right) & =\tau_{k}^{C,a}\left(t\right)+\tau_{k}^{U,a,b}\left(t\right)+\tau_{k}^{C,b}\left(t\right)\\ & +\tau_{k}^{U,b,c}\left(t\right)+\tau_{k}^{C,c}\left(t\right)+\tau_{k}^{G,c}\left(t\right)\\ & +\tau_{k}^{D,c,b}\left(t\right)+\tau_{k}^{G,b}\left(t\right)+\tau_{k}^{D,b,a}\left(t\right)+\tau_{k}^{G,a}\left(t\right). \end{array}$$

Moreover, since the value of 0–1 variable $a_{k,s}\left(t\right)$ is related to the successful participation in training, we can set the maximum training latency $\tau^{\max}\left(t\right)$ to avoid the existence of equipment with extremely poor latency performance, i.e.,

(17) $$z_{k}\left(t\right)=\left\{\begin{array}{c} 1,\;\tau_{k}\left(t\right)\leq \tau^{\max}\left(t\right),\\ 0,\;\tau_{k}\left(t\right)>\tau^{\max}\left(t\right), \end{array}\right.$$

and the actual device–server participation variable is $a_{k,s}\left(t\right)=x_{k,s}\left(t\right)z_{k}\left(t\right)$. This separation removes the circular dependence between association selection and latency-feasible participation.

We can observe that the model splitting strategy and the device association strategy can directly affect the defined training latency, which is affected by multi-dimensional factors such as bandwidth, transmit power, local computing power, and interference. Meanwhile, enhancing learning performance constitutes one of the primary objectives of SD-HFSL. Nevertheless, the influence of optimizing training latency on learning performance warrants additional investigation.

### 2.3. Convergence Analysis

We conducted the convergence analysis of SD-HFSL to quantify the impact of training latency and considered the following three standard assumptions on the loss functions, which are consistent with the FL literature [27,30]. These assumptions are adopted to obtain a tractable upper bound for the semi-decentralized split aggregation process in resource-constrained IoT networks. They are standard in FL convergence analysis and allow us to explicitly connect device participation, inter-edge aggregation reliability, and the loss bound.

**Assumption** **1.** *Each gradient $g_{k}\left(w\right)$ of the local loss $f_{k}\left(w\right)$ is Lipschitz continuous with a positive constant L, i.e., $∥g_{k}\left(w\right)-g_{k}\left(w^{\prime }\right)∥\;\leq L∥w-w^{\prime }∥$. $\forall w,w^{\prime }$.*

**Assumption** **2.** *Each local loss $f_{k}\left(w\right)$ is strongly convex with a positive constant μ, i.e., $f_{k}\left(w\right)\geq f_{k}\left(w^{\prime }\right)+{\left(w-w^{\prime }\right)}^{T}g_{k}\left(w^{\prime }\right)+\frac{\mu }{2}{∥w-w^{\prime }∥}^{2}$, $\forall w,w^{\prime }$.*

**Assumption** **3.** *Each local gradient $g_{k}\left(w\right)$ satisfies $\xi_{1}^{2}\leq {∥g_{k}\left(w\right)∥}^{2}\leq \xi_{2}^{2}$ with $\xi_{1},\xi_{2}>0$, ∀w.*

**Theorem** **1.** *Denote the global optimal model as $w^{*}$, then the upper bound of the training loss on edge server s can be expressed as*

(18) $$\begin{array}{cc} \; & E(\tilde{F_{s}}\left({\tilde{w}}_{s}\left(t+1\right)\right)-{\tilde{F}}_{s}\left(w^{*}\right))\\ & \leq \left(1-{\left(1-\frac{\mu }{L}\right)}^{t+1}\right)\frac{4L\xi_{2}^{2}\left(\sum_{k\in K}D_{k}-NU^{\min}\right)}{\mu \sum_{k\in K}D_{k}}\\ & +{\left(1-\frac{\mu }{L}\right)}^{t+1}E\left(F_{s}\left({\bar{w}}_{s}\left(1\right)\right)-F_{s}\left(w^{*}\right)\right), \end{array}$$

*where ${\tilde{w}}_{s}\left(t\right)=\frac{\sum_{k\in V_{s}\left(t\right)}D_{k}w_{k}\left(t\right)}{\sum_{k\in V_{s}\left(t\right)}D_{k}}$. Here, $u_{k}^{D}\left(t\right)$ denotes the probability that device k successfully participates in the current round under the latency budget, $u_{s,s^{\prime }}^{E}\left(t\right)$ denotes the reliability of inter-edge model exchange from edge server $s^{\prime }$ to s, and NU(t) denotes the amount of successfully aggregated data, including both intra-cluster and inter-cluster contributions.*

(19) $$\begin{array}{cc} \; & NU^{\min}=\min_{t\in T}NU\left(t\right)\\ & =\min_{t\in T}\sum_{k\in V_{s}\left(t\right)}u_{k}^{D}\left(t\right)D_{k}+\sum_{s^{\prime }\in C_{s}\left(t\right)}\sum_{k^{\prime }\in V_{s^{\prime }}\left(t\right)}u_{k^{\prime }}^{D}\left(t\right)u_{s,s^{\prime }}^{E}\left(t\right)D_{k^{\prime }}. \end{array}$$

According to Theorem 1, increasing the device participation probability or increasing the reliability of communication between edge servers in $NU^{\min}$ can reduce the upper bound of training loss (see Appendix A). Moreover, it is worth noting that the factors affecting the learning performance among devices are not only the amount of locally owned data, but also the convergence parameters, such as *L* and μ. Specially, we define the local gradient of device *k* as $g_{k}\left(t\right)$ rather than $g_{k}\left(\zeta_{k}^{j}\left(t\right),w_{k}^{\left(j-1\right)}\left(t\right)\right)$, which can represent the average gradient or the gradient based on local complete data. This simplification has little effect on the results of the analysis, since this paper focus on the cost of edge servers rather than the local updates among the devices. In particular, the data held by the devices that do not have a low local loss based on the current edge model should be more important for improving learning performance. Finally, in light of the analytical findings of the convergence performance, we may rebuild the objective function using the acquired upper limit. It should be emphasized that the above theorem provides theoretical insight under simplified convex assumptions. The DNNs used in the experiments, such as AlexNet, VGG16, and ResNet18, are non-convex. Therefore, the bound should not be interpreted as a complete convergence guarantee for these neural networks. Instead, it explains why reducing latency and increasing latency-feasible participation are useful design principles. The experimental results then provide empirical evidence that the same principles improve SD-HFSL performance in practical non-convex training.

## 3. Problem Formulation

The considered problem for SD-HFSL is a sequential decision-making problem, and the goal is to maximize the training efficiency. Specifically, to jointly optimize the learning performance and training latency, the training efficiency can be evaluated as the decrease in training losses per unit of time. Hence, for each device *k*, the training efficiency can be defined as

(20) $$u_{k}\left(t\right)=\sum_{s\in S}\frac{a_{k,s}\left(t\right)\left({\tilde{F}}_{s}\left({\tilde{w}}_{s}\left(t-1\right)\right)-{\tilde{F}}_{s}\left({\tilde{w}}_{s}\left(t\right)\right)\right)}{\tau_{k}\left(t\right)},$$

where ${\tilde{F}}_{s}\left({\tilde{w}}_{s}\left(t-1\right)\right)-{\tilde{F}}_{s}\left({\tilde{w}}_{s}\left(t\right)\right)$ indicates the reduction of training loss under the associated edge model.

Then, the system utility to represent the training efficiency can be expressed as

(21) $$U\left(t\right)=\sum_{k\in K}u_{k}\left(t\right).$$

Different from common optimization problems, this paper considers the unknown randomness in local computing power changes, resulting in training latency is uncertain in different states of devices, which can be evaluated based on the context information $\hat{C}\left(t\right)$, including channel state, allocated bandwidth, transmit power, interference, etc. Then, the formulation of the online maximization problem with model splitting strategy I and device association strategy X can be expressed as

(22a)max{I,X}∑t=1TU(I(t),X(t),C^(t))(22b)s.t.1≤Ika,b(t)≤Imax−2,∀k∈K,t∈T,(22c)Ika,b(t)<Ikb,c(t)≤Imax−1,∀k∈K,t∈T,(22d)xk,s(t)∈{0,1},∀k∈K,∀s∈S,t∈T,(22e)∑s∈Sxk,s(t)≤1,∀k∈K,t∈T,(22f)∑k∈Kxk,s(t)≤Asmax,∀s∈S,t∈T,

where the model splitting strategy is coupled with the device association strategy, the maximum number of devices associated with edge server *s* is defined as $A_{s}^{\max}$, and each device can be associated with, at most, one edge server. The latency-feasible variable $z_{k}\left(t\right)$ and the effective participation variable $a_{k,s}\left(t\right)=x_{k,s}\left(t\right)z_{k}\left(t\right)$ are then evaluated after the candidate association and split decision are determined.

There are two challenges to be addressed to solve this online maximization problem. On the one hand, precise training latency estimation is necessary for the model splitting decision to improve the expected utility when implemented. Since the system evaluates long-term performance and is based on cold starts, it cannot utilize historical information and can only continuously estimate the training latency of the device from multiple rounds. On the other hand, the relationship between training loss and optimization variables in the utility function can not accurately describe the effect of the splitting strategy on learning performance.

To solve these issues, we examine the convergence of the introduced framework under the previous analysis results in [10]. Specially, with limited latency budget $\tau^{\max}$, allowing more devices to participate in training can accelerate the convergence rate of training loss, i.e.,

(23) $$\begin{array}{cc} \; & {\tilde{F}}_{s}\left({\tilde{w}}_{s}\left(t-1\right)\right)-{\tilde{F}}_{s}({\tilde{w}}_{s}\left(t\right))\propto \\ & \;\sum_{k\in V_{s}\left(t\right)}a_{k,s}\left(t\right)D_{k}\varpi^{2}+\sum_{s^{\prime }\in C_{s}\left(t\right)}\sum_{k^{\prime }\in V_{s^{\prime }}\left(t\right)}a_{k^{\prime },s^{\prime }}\left(t\right)D_{k^{\prime }}\varpi^{2}, \end{array}$$

where $\varpi^{2}\geq {∥g_{k}\left(w\right)∥}^{2}$ is the upper bound of the local gradient. Equation (23) uses the effective participation variable $a_{k,s}\left(t\right)$ and therefore counts the devices that successfully contribute to the model update, while the residual term in Theorem 1 is reduced when this successful data mass increases.

Then, the right side of the proportional Formula (23) is brought into the utility function (21) and is transformed into a problem that can be solved with a deterministic solution.

## 4. Joint Model Splitting and Device Association

We assume that only splitting and association strategies related to transmission latency can be further optimized, while other parameters, such as bandwidth allocation, channel gain, transmit power, and co-frequency interference are predetermined as the context information of the current training round. Specifically, we formulate the proposed long-term model splitting problem as a CMAB with online decisions. The strategy is “online” because the training latency among devices and edge servers is unknown in advance, and is evaluated based on the context associated with the system. Moreover, given the estimated latency information, we propose a joint splitting and association algorithm to further improve system utility.

### 4.1. Online Latency Estimation Based on CMAB

In CMAB, assuming that the existing control center can observe the context of the system at the beginning of each training round before performing model updates. Let $c_{k}\left(t\right)\in c\left(t\right)$ be the context of device *k* observed in training round *t*, where c(t) is the context space and can be limited by quantitative coding. Similarly, we denote the context of edge server *s* as ${\tilde{c}}_{s}\left(t\right)\in c\left(t\right)$. Then, the context of the system is collected in

(24) $$\begin{array}{cc} c(t)= & \{c_{1}\left(t\right),c_{2}\left(t\right),\ldots ,c_{K}\left(t\right),{\tilde{c}}_{1}\left(t\right),{\tilde{c}}_{2}\left(t\right),\ldots ,{\tilde{c}}_{S}\left(t\right)\}. \end{array}$$

The local computing power $\left\{f_{k}\left(t\right),{\tilde{f}}_{s}\left(t\right)\right\}$ of device *k* and edge server *s* is a random variable parameterized by the context $\left\{c_{k}\left(t\right),{\tilde{c}}_{s}\left(t\right)\right\}$. Hence, we can rewrite the computing power vector in a context-aware form, i.e.,

(25) $$\begin{array}{cc} f(t)= & \{f_{1}\left(c_{1}\left(t\right)\right),f_{2}\left(c_{2}\left(t\right)\right),\ldots ,f_{K}\left(c_{K}\left(t\right)\right),\\ & {\tilde{f}}_{1}\left({\tilde{c}}_{1}\left(t\right)\right),{\tilde{f}}_{2}\left({\tilde{c}}_{2}\left(t\right)\right),\ldots ,{\tilde{f}}_{S}\left({\tilde{c}}_{S}\left(t\right)\right)\}. \end{array}$$

Moreover, let $\psi_{k}\left(c_{k}\left(t\right)\right)≜E\left[f_{k}\left(t\right)|c_{k}\left(t\right)\right]$ and ${\tilde{\psi }}_{s}\left({\tilde{c}}_{s}\left(t\right)\right)≜E\left[{\tilde{f}}_{s}\left(t\right)|{\tilde{c}}_{s}\left(t\right)\right]$ be the expected values of the unknown local computing power of device *k* and edge server *s*, respectively. Then, the vector of the expected local computing power is given by

(26) $$\begin{array}{cc} \psi (t)= & \{\psi_{1}\left(c_{1}\left(t\right)\right),\psi_{2}\left(c_{2}\left(t\right)\right),\ldots ,\psi_{K}\left(c_{K}\left(t\right)\right),\\ & {\tilde{\psi }}_{1}\left({\tilde{c}}_{1}\left(t\right)\right),{\tilde{\psi }}_{2}\left({\tilde{c}}_{2}\left(t\right)\right),\ldots ,{\tilde{\psi }}_{S}\left({\tilde{c}}_{S}\left(t\right)\right)\}. \end{array}$$

The core of the CMAB approach is the use of contextual information c(t) and computing power f(t) to simulate the actual training latency. In practice, the control center does not have a priori knowledge of the training latency affected by the local computing power. Hence, the joint splitting and association strategy is optimized based on the estimation results, which can be replaced by ψ(t) in each training round. More specifically, the context is the quantized vector c(t), the action is the joint decision {I(t),X(t)}, and the reward is the system utility U(t) defined in (21). The oracle policy is the policy that knows the expected computing capability ψ(t) for every context and selects the best feasible joint decision in each round.

The context-aware online optimization algorithm designed in the framework of CMAB is executed simultaneously with the model training process. In each time slot or training round *t*, sequential decision-making is performed at a hypothetical control center as follows: (1) The control center observes the context set c(t) of all devices and edge servers. (2) The control center determines the model splitting decision I(t) and the device association strategy X(t) based on the observed latest computing power information ψ(t) in the current training round. (3) The optimized strategy is applied for model training, where if $I_{k}^{a,b}\left(t\right)=I_{k}^{b,c}\left(t\right)=0$, and device *k* can update its local model locally with complete model transfer to the associated edge servers; otherwise, three-part training is performed. (4) At the end of each training round, the actual local computing power among devices and edge servers is observed, which is then used to update the computing power estimation ψ(t) with the observed context c(t). Especially, if the latency budget cannot be satisfied, the local computation time concerned is upper-bounded to meet $\tau_{k}\left(t\right)=\tau^{\max}\left(t\right)$.

To make the context-aware demand estimation tractable, the context space scope needs to be designed to be limited. To be specific, the system context c(t) can be divided into small hypercubes with context partition. Meanwhile, the state of each device or edge server in different training rounds will be different, especially considering the complex communication environment and interference that is difficult to predict in advance. Therefore, the computing power estimation process needs to be performed separately on each device and edge server with fine-grained induction. Then, a key issue is estimating the local computing power pattern for context hypercubes at each device or each edge server. The proposed online optimization algorithm runs with a cold start, where local computing power can only be estimated based on the context of the hypercubes observed in each training round with an accumulation of historical information. In particular, to estimate the number of times that device *k* or edge server *s* contributes local computing power up to training round *t*, the control center has two types of counters $O_{k}\left(c_{k}\left(t\right)\right)$ and ${\tilde{O}}_{s}\left({\tilde{c}}_{s}\left(t\right)\right)$, with different contexts. The control center also keeps two experiences, $E_{k}\left(c_{k}\left(t\right)\right)$ and ${\tilde{E}}_{s}\left({\tilde{c}}_{s}\left(t\right)\right)$, from hypercube in training round *t* to store the context-power pair {c(t),f(t)}. Then, given the experiences, the local computing power estimated for device *k* and edge server *s* are obtained by estimator θ, i.e.,

(27) $$\psi_{k}\left(c_{k}\left(t\right)\right)=\theta \left(E_{k}\left(c_{k}\left(t\right)\right),O_{k}\left(c_{k}\left(t\right)\right)\right),$$

and

(28) $${\tilde{\psi }}_{s}\left({\tilde{c}}_{s}\left(t\right)\right)=\theta \left({\tilde{E}}_{s}\left({\tilde{c}}_{s}\left(t\right)\right),{\tilde{O}}_{s}\left({\tilde{c}}_{s}\left(t\right)\right)\right),$$

respectively. In particular, in this paper, we consider an unbiased estimation θ(·) using maximum likelihood estimation, i.e.,

(29) $$\psi_{k}\left(c_{k}\left(t\right)\right)=\frac{\sum_{c\in E_{k}\left(c_{k}\left(t\right)\right)}f_{k}\left(c\right)}{O_{k}\left(c_{k}\left(t\right)\right)},$$

and

(30) $${\tilde{\psi }}_{s}\left({\tilde{c}}_{s}\left(t\right)\right)=\frac{\sum_{c\in {\tilde{E}}_{s}\left({\tilde{c}}_{s}\left(t\right)\right)}{\tilde{f}}_{s}\left(c\right)}{{\tilde{O}}_{s}\left({\tilde{c}}_{s}\left(t\right)\right)}.$$

Algorithm 1 illustrates the pseudo code of the online optimization algorithm.

Specifically, in each training round *t*, the algorithm is in either an exploration phase or an exploitation phase. To determine the phase, the most important criterion is whether the local computing power corresponding to the current context information is fully explored. Hence, the sets of under-explored devices and edge servers are denoted as $K^{\mathrm{ue}}\left(t\right)=\left\{k\in K,O_{k}\left(c_{k}\left(t\right)\right)\leq O_{k}\left(t\right)\right\}$ and $S^{\mathrm{ue}}\left(t\right)=\left\{s\in S,{\tilde{O}}_{s}\left({\tilde{c}}_{s}\left(t\right)\right)\leq {\tilde{O}}_{s}\left(t\right)\right\}$, respectively, where $O\left(t\right)=\{O_{k}\left(t\right),{\tilde{O}}_{s}\left(t\right)\}$ is a threshold set to determine whether the historical data of devices and edge servers can enter the exploitation stage. In particular, since the training latency affected by the local computing power is the final expression in the proposed problem, we can evaluate the training latency based on the joint splitting and association strategy indirectly. In addition, since the combination of device k∈K and edge server s∈S can be viewed as an arm, according to the sets of under-explored devices and edge servers, the procedures of exploration and exploitation are as follows.

(1) *Exploration* If the under-explored set is non-empty, device $k\in V_{s}\left(t\right)$ and its associated edge server *s* in the under-explored set have two cases. In case 1, for edge server *s*, the number of devices associated with the current training round satisfies $\sum_{k\in K^{\mathrm{ue}}}a_{k,s}\left(t\right)=A_{s}^{\max},\exists s\in S$, where the remaining devices $k\in K^{\mathrm{ue}}\left(t\right)/V_{s}\left(t\right)$ in the collection can be removed from the association policy in the current arm. In case 2, when the under-explored devices and edge servers satisfy $\sum_{k\in K^{\mathrm{ue}}}a_{k,s}\left(t\right)<A_{s}^{\max},\forall s\in S$. In this case, the devices can greedily associate existing edge servers in order.Meanwhile, since we have defined the latency budget $\tau^{\max}$, the latency that exceeds the budget during the exploration process is always denoted as $\tau_{k}=\tau^{\max}$.
**Algorithm 1** Context-Aware Online Optimization Algorithm  1: **Input:** K, S, *T*, context partition, exploration thresholds, and latency budget $\tau^{\max}$.  2: **Output:** online splitting and association decisions ${\left\{I\left(t\right),X\left(t\right)\right\}}_{t=1}^{T}$.  3: **Initialization**. initialize $O_{k}\left(c_{k}\left(t\right)\right)=0$, ${\tilde{O}}_{s}\left({\tilde{c}}_{s}\left(t\right)\right)=0$, ∀k,s,t, and choose an estimator θ based on maximum likelihood estimation.  4:  for t=1,2,…,T do  5:    Observe the context c(t) and counter O(t)  6:    Evaluate under-explored devices and edge servers  7:    if $K^{\mathrm{ue}}\left(c\left(t\right)\right)\cup S^{\mathrm{ue}}\left(c\left(t\right)\right)\neq \emptyset$ then ▷ Exploration  8:    else  9:    Solving the problem in (22) ▷ Exploitation10:    end if11:    for each devices $k\in K^{\mathrm{ue}}\left(t\right)$ do12:   Update counter $O_{k}\left(c_{k}\left(t\right)\right)=O_{k}\left(c_{k}\left(t\right)\right)+1$13:   Update estimations $\psi_{k}\left(c_{k}\left(t\right)\right)=\frac{\sum_{c\in E_{k}\left(c_{k}\left(t\right)\right)}f_{k}\left(c\right)}{O_{k}\left(c_{k}\left(t\right)\right)}$14:   Update experiences $E_{k}\left(c_{k}\left(t\right)\right)=E_{k}\left(c_{k}\left(t\right)\right)\cup \left\{c_{k}\left(t\right),\psi_{k}\left(c_{k}\left(t\right)\right)\right\}$15:    end for16:    for each edge server $s\in S^{\mathrm{ue}}\left(t\right)$ do17:   Update counter ${\tilde{O}}_{s}\left({\tilde{c}}_{s}\left(t\right)\right)={\tilde{O}}_{s}\left({\tilde{c}}_{s}\left(t\right)\right)+1$18:   Update estimations ${\tilde{\psi }}_{s}\left({\tilde{c}}_{s}\left(t\right)\right)=\frac{\sum_{c\in {\tilde{E}}_{s}\left({\tilde{c}}_{s}\left(t\right)\right)}{\tilde{f}}_{s}\left(c\right)}{{\tilde{O}}_{s}\left({\tilde{c}}_{s}\left(t\right)\right)}$19:   Update experiences ${\tilde{E}}_{s}\left({\tilde{c}}_{s}\left(t\right)\right)={\tilde{E}}_{s}\left({\tilde{c}}_{s}\left(t\right)\right)\cup \left\{{\tilde{c}}_{s}\left(t\right),{\tilde{\psi }}_{s}\left({\tilde{c}}_{s}\left(t\right)\right)\right\}$20:    end for21:   Perform joint model splitting and device association strategy22:  end for23:  **Return** {I(t),X(t)}.

(2) *Exploitation*: If the explored set is non-empty, we can optimize the joint problem of model splitting and device association strategy based on the current estimated value ψ(t). Finally, given the feedback latency information, the counter, estimations, and experiences are also updated. Then, the model splitting problem can be decoupled from the device association problem, i.e.,

(31a)  max{I(t)}U(X∗(t),C^(t))(31b)s.t. (22b), (22c),

and

(32a)  max{X(t)}U(I∗(t),C^(t))(32b)s.t. (22d)–(22f),

respectively. Hence, given a fixed device association strategy, the optimal model splitting can be evaluated based on the exhaustive search. Since the model splitting strategy between the device and the edge server is carried out independently, we can infer that the complexity for the problem (31) is $O({\left(KI^{\max}\right)}^{2})$. Meanwhile, given the fixed model splitting strategy, the evaluated training latency among devices and edge servers can be evaluated. Then, a utility matching matrix H, including the latency from the devices to the edge servers, is designed. Since each edge server can associate multiple devices, the original association is a many-to-one assignment. To use the KM algorithm, edge server *s* is expanded into $A_{s}^{\max}$ virtual server slots, each of which has the same utility weight as device *k*. The resulting matrix has *K* device nodes and $\sum_{s\in S}A_{s}^{\max}$ virtual slots. A one-to-one matching over the virtual slots is therefore equivalent to the original capacity-constrained association because each device is matched to, at most, one slot, and no edge server receives more than $A_{s}^{\max}$ devices because it owns only $A_{s}^{\max}$ slots. If the two sides have different cardinalities, dummy nodes with zero utility are added to obtain a square matrix.

For a device–slot pair corresponding to edge server *s*, the matching weight is defined as the estimated utility, e.g., $1/\tau_{k}$ or the latency-normalized utility in (21), after the split decision is fixed.

The model splitting strategy and device association strategy are performed iteratively until the objective function is convergent, where the convergence criterion for a given threshold ϵ is defined as $U\left(X^{*}\left(t\right),\hat{C}\left(t\right)\right)-U\left(I^{*}\left(t\right),\hat{C}\left(t\right)\right)\leq \varepsilon$ and the strategies in each iteration *j* are denoted as $I^{\left(j\right)}\left(t\right)$ and $X^{\left(j\right)}\left(t\right)$, respectively.

Moreover, the performance of online decision-making is measured by utility loss, termed regret, compared to the utility achieved by the oracle policy. Let $U^{★}\left(c\left(t\right)\right)$ be the expected utility achieved by the oracle policy under context c(t), and let U(t) be the utility achieved by the proposed online policy. The cumulative regret is

(33) $$\begin{array}{c} R\left(T\right)=\sum_{t=1}^{T}\left(U^{★}\left(c\left(t\right)\right)-U\left(t\right)\right). \end{array}$$

Under bounded utility and a finite context partition, the regret consists of exploration regret and exploitation regret. For |C| context hypercubes and an exploration threshold growing as $O(t^{\rho }\log t)$, 0<ρ<1, the number of forced exploration rounds is sublinear in *T*. The exploitation regret is also sublinear because the sample-mean estimates of the context-dependent computing capabilities converge to their expectations. Following the standard finite-partition CMAB concentration argument in [38], which bounds forced exploration and estimation error separately, the expected regret is upper bounded by

(34) $$\begin{array}{c} E\left[R\left(T\right)\right]\leq C_{1}\left(K+S\right)\left|C\right|T^{\rho }\log T+C_{2}T^{1-\rho /2}, \end{array}$$

where $C_{1}$ and $C_{2}$ are constants related to the bounded utility range and estimation error. Thus, E[R(T)]/T→0 as T→∞, which indicates sublinear regret. Larger *K*, *S*, context dimension, or finer context partition increases the constants and slows convergence, which is also observed in the simulations.

### 4.2. Complexity Optimization for Online Learning

It is worth noting that the complexity of the KM algorithm in (32) is $O(\max{\left\{K,S\right\}}^{3})$, thus, it is more complicated to use the KM algorithm in each exploitation stage. To solve this issue, as shown in Algorithm 2, we can use a simple greedy algorithm with complexity O(K) to solve device association in the first few exploitation stages. Then, a joint optimization algorithm is adopted when the estimates are stabilized. In particular, we set a threshold κ, and if t≤κ, we allow each device to be independently associated with the edge server based on its optimal training latency until the maximal association constraint is satisfied. Moreover, even if the model splitting strategy can be selected among the limited optimization results, the complexity of the iterative optimization strategy can not be ignored. To solve this issue, considering the model splitting process, according to the transmission and computing processes, the model splitting strategy for exploration can be divided into two cases. (1) The device completes the model training locally without model splitting. (2) Only the first and last layer neural networks are trained locally at the device, and the remaining layers are trained at the edge server. Finally, according to the estimated local computing power of the current round, a better model splitting strategy for exploration is chosen from the above two cases to achieve lower complexity.
**Algorithm 2** Joint Model Splitting and Device Association Algorithm  1:  **Input:** K, S, *t*, $\hat{C}\left(t\right)$, $\psi_{k}\left(c_{k}\left(t\right)\right)$, ${\tilde{\psi }}_{s}\left({\tilde{c}}_{s}\left(t\right)\right)$, $A_{s}^{\max}$, κ, and ε.  2:  **Output:** splitting strategy $I^{*}\left(t\right)$ and association strategy $X^{*}\left(t\right)$.  3:  **Initialization**. j=0, I(t), X(t).  4:  **Repeat**.  5:    if t≥κ  6:      for k=1,2,…,K  7:        Ergodic search $I_{k}^{a,b}\left(t\right)$ and $I_{k}^{b,c}\left(t\right)$ with fixed association strategy $X^{*}\left(t\right)$ and obtain the temporary splitting strategy $I^{*}\left(t\right)=I^{\left(j\right)}\left(t\right)$  8:      else  9:        Perform model splitting from two fixed strategies10:      end for11:  Update the current latency matching matrix H12:  if t≥κ13:      Obtain the association strategy $X^{*}\left(t\right)=X^{\left(j\right)}\left(t\right)$ based on KM algorithm with fixed model splitting strategy $I^{*}\left(t\right)$14:    else15:      Obtain the association strategy $X^{*}\left(t\right)=X^{\left(j\right)}\left(t\right)$ based on greedy algorithm with fixed model splitting strategy $I^{*}\left(t\right)$16:    end if17:    j=j+118:  Until $U\left(X^{*}\left(t\right),\hat{C}\left(t\right)\right)-U\left(I^{*}\left(t\right),\hat{C}\left(t\right)\right)\leq \varepsilon$.19:  **Return**. $I^{*}\left(t\right)$, $X^{*}\left(t\right)$.

## 5. Numerical Results

In this section, we present the details of the simulation results and evaluate the performance of the proposed SD-HFSL based on the framework of CMAB [41].

### 5.1. Experiment Settings

For the simulation results, we assume that there are S=5 edge servers and K=25 devices (if not specified) randomly located in a square area with the size of 500m×500m. Moreover, each edge server can associate at most with five devices, i.e., $A_{s}^{\max}=5,\forall s\in S$. In addition, the context space has two dimensions, namely “*task workload of edge servers*” and “*wireless communication environment*”. For instance, the context “*task workload of edge servers*” indicates the case where the devices and edge servers take on other computing tasks, and only a part of local computing power can be used for training. The “*wireless communication environment*” is the communication rate between devices and edge servers. For reproducibility, the wireless links use distance-dependent path loss with small-scale fading, the transmit powers of devices and edge servers are set to 23 dBm and 30 dBm, respectively, the total bandwidth is 20 MHz and equally divided among active links, and the noise power spectral density is −174 dBm/Hz. All reported curves are averaged over 20 independent trials with random locations, computing powers, and non-IID data partitions. Across these repeated trials, the result variation is small. The relative standard deviation of the latency and utility curves is within about 2.5% of the mean, and the 95% confidence interval of the final test accuracy is within about ±0.4 percentage points. Since each figure contains multiple baseline curves, these narrow intervals are visually difficult to distinguish and would reduce readability. This behavior is different from reinforcement learning training curves, where policy exploration often causes much wider fluctuations. Then, to describe the unknown local computing power under different contexts, we can assume that the local computing power of device *k* and edge server *s* is uniformly distributed in the interval $\left[f_{k}^{\min}\left(c\left(t\right)\right),f_{k}^{\max}\left(c\left(t\right)\right)\right]$ $\left[{\tilde{f}}_{s}^{\min}\left(c\left(t\right)\right),{\tilde{f}}_{s}^{\max}\left(c\left(t\right)\right)\right]$, respectively, where the actual computing power of the device *s* is on the order of $10^{9}$ cycles/second, and the actual computing power of the edge server *s* is usually on the order of $10^{11}$ cycles/second, and the upper and lower bounds of computing power vary with the context information. In particular, to reduce the complexity of simulation, we consider four contextual situations that occur at equal intervals over 1000 training rounds, and for each device and edge server pair, the elements in the threshold set O(t) are set as 50. Moreover, for the learning task, we consider non-i.i.d. local training data among devices and conduct the experiments using the CIFAR-10 datasets. By default, we train the learning model based on AlexNet, which consists of five convolutional layers, three fully connected layers, and finally the Softmax classification layer, where the batch size is set to 64, and the learning rate is set to 0.02. The number of model splitting points is $I^{\max}=8$. Moreover, to simulate the imbalance of the number distribution, we consider that each device has only two types of training samples. The experimental data setup and the main parameters are summarized in Table 3. These values are selected to emulate resource-constrained IoT devices assisted by stronger edge servers. The latency budget and exploration threshold are set to make the system experience both successful and unsuccessful participation events, which is necessary to evaluate online adaptation under incomplete information.

Finally, the proposed algorithm is compared with the following five benchmarks: (1) Oracle algorithm. The latency values between devices and edge servers are known in advance, and the optimal latency is evaluated when all devices can be successfully associated. (2) Greedy algorithm. Based on the existing experience, devices are associated with the edge server with the lowest training latency and set κ=T; that is, the KM algorithm is not used to optimize the association strategy. (3) UCB algorithm. The expected utility of each local computing power is evaluated over time by enumerating all splitting and association decisions without considering the context information. Moreover, when the association constraint is not satisfied, devices randomly select the edge server. (4) Random. Each device is randomly associated with the edge server. When the edge server association constraint is not satisfied, the device with large training latency is removed. (5) Noncooperative algorithm. Regardless of model splitting, devices perform model training locally. The proposed method also has limitations. It requires enough repeated observations in each context hypercube to obtain reliable estimates, and the matching step may become costly when the number of devices and virtual edge-server slots is very large. In addition, the present simulations focus on latency and learning efficiency rather than attacks on exchanged features or gradients.

### 5.2. Results and Discussions

In Table 4, to intuitively reflect the impact of the model splitting strategy on training latency, we randomly select a device *k* in training round *t*, and define its splitting strategy $I_{k}^{a,b}\left(t\right)$ and $I_{k}^{b,c}\left(t\right)$ as $I^{1}$ and $I^{2}$, respectively.

From Table 4, we can observe that when there is an obvious difference between the device’s local computing power and the edge server, the device tends to send as many computing tasks as possible to the edge server to reduce the training latency. Specifically, considering the parameter distribution of AlexNet, the training latency can increase significantly when the splitting strategy $I^{1}\geq 2$ due to the large computational amount of the second convolution layer. Moreover, because of the maximum training budget $\tau^{\max}$, device *k* is unable to successfully participate in training in this case. Hence, the splitting strategy has a direct impact on the training latency and the number of devices participating in the training, and needs to be fully optimized.

**Figure 2:**
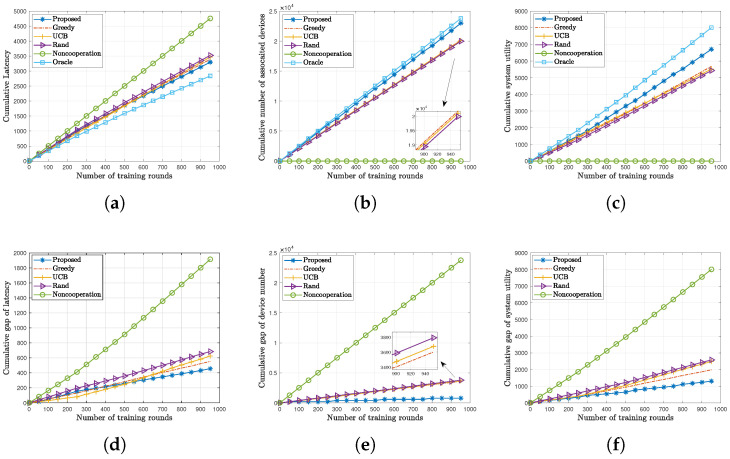
Comparison on cumulative algorithm performance. (**a**) Cumulative training latency. (**b**) Cumulative number of associated devices. (**c**) Cumulative system utility. (**d**) Cumulative gap of latency. (**e**) Cumulative gap of device number. (**f**) Comparison gap of system utility.

Figure 2 shows the cumulative performance of the proposed algorithm and the baselines. In particular, in Figure 2a, we compare the cumulative training latency $\sum_{t=1}^{T}\tau \left(t\right)$ of different algorithms. The results show that, except for the oracle and greedy algorithms, the proposed algorithm can obtain the lowest training latency. In addition, it is interesting that the proposed algorithm has no obvious advantage over the greedy algorithm and the UCB algorithm in the initial training rounds. This is because, to reduce the complexity, the proposed algorithm also completes device association in a greedy way under the exploration phase and has the same execution process as the comparison algorithms. Specifically, when the KM algorithm is taken into account, the advantages of the proposed algorithm in different contexts are constantly amplified. In Figure 2b, we compare the cumulative number of participating devices $\sum_{t=1}^{T}\sum_{k\in K}\sum_{s\in S}a_{k,s}\left(t\right)$. We can observe that our proposed algorithm is also the best, except for the oracle algorithm, due to the fact that we can realize the maximum total matching by the KM algorithm in the utilization phase. Other comparison algorithms cannot deal with the maximum number of association constraints, so more devices cannot participate in the training when the association strategy conflicts. Moreover, in Figure 2c, we evaluate the cumulative system utility $\sum_{t=1}^{T}\frac{\sum_{k\in K}\sum_{s\in S}a_{k,s}\left(t\right)}{\tau (t)}$, considering the training latency and the number of the participating devices. The proposed algorithm obtains a higher cumulative utility than the non-oracle baselines. In addition, in order to further reflect the gap between the proposed algorithm and the optimal strategy, we define the cumulative inability; that is, we calculate the difference between the proposed algorithm and the oracle algorithm in the training latency, the number of participating devices, and the system utility. In Figure 2d–f, it can be seen that after full exploration, the training latency and the system utility of the proposed algorithm have the slowest growing trend, while the number of devices participating in training is almost consistent with the optimal case, which can demonstrate the effectiveness of the proposed context-based estimation strategy.

**Figure 3:**
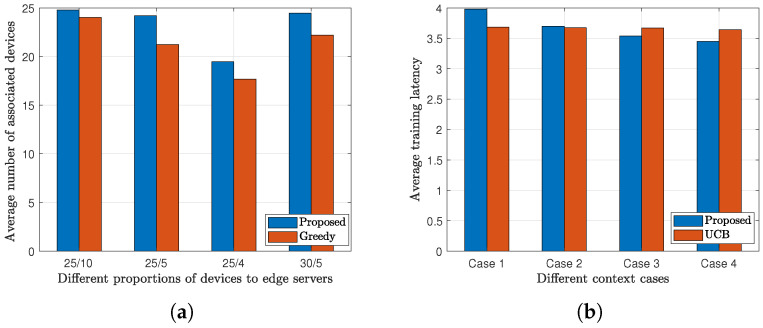
Performance under different experiment settings. (**a**) Average number of associated devices with different proportions of devices and edge servers. (**b**) Average training latency with different context types.

Moreover, to further explain the reasons for the advantages of our proposed algorithm, we especially compare the proposed algorithm with the greedy and UCB algorithms. Specifically, in Figure 3a, we changed the proportions of devices to edge servers while evaluating the average number of associated devices in training, which can be denoted as $\frac{\sum_{t=1}^{T}\sum_{k\in K}\sum_{s\in S}a_{k,s}\left(t\right)}{T}$. We can observe that when the number of devices is small, i.e., K=25, and the number of edge servers is large, i.e., S=10, more devices can be associated with the ideal edge server, and there is little difference between the proposed algorithm and the greedy algorithm. On the contrary, when the number of terminals is large, i.e., K=30, there is an upper bound on the maximum number of associations due to the limit of the maximum number of devices associated in a single edge server, and there is no significant change as the number of devices increases. In addition, we further compare the average training latency $\frac{\sum_{t=1}^{T}\tau \left(t\right)}{T}$ of the proposed algorithm and the UCB algorithm under different numbers of context types. In particular, we assume that the larger the context index, the longer the training latency of a single training round. Moreover, different contextual situations occur in order per each 250 training rounds. Then, the results show that when the context type is small, the performance of the proposed algorithm is close to that of the UCB algorithm, and the advantages of the proposed algorithm are more significant with the increase of the context types. This is because our proposed algorithm considers the context when estimating training latency, and dynamically updates the model splitting and association strategies under different context backgrounds to improve performance.

**Figure 4:**
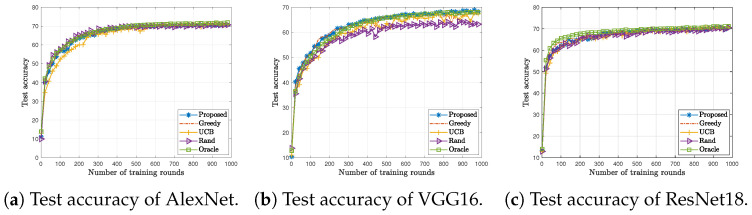
Learning performance under different neural network structures and different numbers of training rounds.

Finally, based on the above simulation results, we also trained both VGG16 and ResNet18 networks in the process of verifying learning performance. Specifically, VGG16 consists of 13 convolutional layers and three fully connected layers, which are separated by the maximization pool, and the activation units of all hidden layers use ReLU functions. Moreover, the network structure of ResNet18 consists of 17 convolutional layers and a final fully connected layer, where each basic block consists of two convolution layers and a skip connection. In Figure 4, we evaluate the learning performance of different neural network structures under different numbers of training rounds. The final test accuracy increases when more devices participate in training.

**Figure 5:**
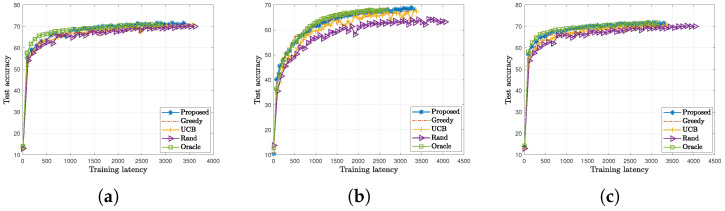
Learning performance under different neural network structures and different training latency. (**a**) Test accuracy based on AlexNet. (**b**) Test accuracy of VGG16. (**c**) Test accuracy of ResNet18.

Moreover, in Figure 5, except for the oracle algorithm, we can observe that the proposed algorithm based on different DNNs can obtain higher test accuracy in the same time frames, especially in the case of VGG16.

Moreover, with the increase in neural network complexity, test accuracy and convergence speed can increase simultaneously. Interestingly, although the computational amount of these neural networks is very different, the training latency in the simulation results does not seem to have a large gap. This is because the local computing capacity of the device is weak, considering that the training latency of the edge server cannot completely offset the local computing latency when the model is split.

## 6. Conclusions

In this paper, we investigated the long-term training efficiency of the SD-HFSL framework. A joint optimization problem of model splitting and device association was formulated based on the convergence performance. Meanwhile, a context-aware online training algorithm was proposed based on the framework of CMAB. We defined the training of the split training process and evaluated the learning performance based on the number of participating devices. The devices and edge servers can observe the context information of device sites for the latency estimation, followed by the iterative optimization based on the evaluated updated information in different contexts. Our experiments used different baselines and model structures to show that the proposed algorithm achieves lower training latency and higher test accuracy in the considered settings, especially when prior latency information is unavailable. Future work will consider privacy protection against feature and gradient leakage and will further evaluate the proposed online algorithm in real-world edge testbeds. Scalable low-complexity matching methods are also needed for larger IoT and 6G networks with fast-varying contexts [42].

## Figures and Tables

**Table 1:** Comparison between related learning frameworks and the proposed SD-HFSL.

Work	Architecture	Multiple Edge Servers	Model Splitting	Device Association	Online/Context Learning	Regret Analysis
HSFL [25]	Centralized hybrid FL-SL	No	Yes	Limited	No	No
RingSFL [26]	Ring-based split FL	No	Yes	No	No	No
Hierarchical split FL [30]	Multi-tier hierarchy	Yes	Yes	Limited	No	No
Semi-asynchronous FSL [31]	Asynchronous edge learning	No	Yes	Client selection	No	No
Semi-decentralized FL [34,35,36]	Edge-to-edge aggregation	Yes	No	Yes	No	No
Proposed SD-HFSL	Semi-decentralized HFSL	Yes	Three-part split	Jointly optimized	Context-aware CMAB	Yes

**Table 2:** Summary of key notation.

Notation	Definition
K,S	Sets of IoT devices and edge servers
$D_{k}$	Number of training samples at device *k*
$I_{k}^{a,b},I_{k}^{b,c}$	Two split points separating parts *a*, *b*, and *c*
$x_{k,s}\left(t\right)$	Association decision between device *k* and edge server *s*
$z_{k}\left(t\right)$	Successful participation indicator under the latency budget
$a_{k,s}\left(t\right)$	Effective participation, i.e., $a_{k,s}\left(t\right)=x_{k,s}\left(t\right)z_{k}\left(t\right)$
$\tau_{k}\left(t\right),\tau \left(t\right)$	Device latency and synchronous round latency
$c_{k}\left(t\right),{\tilde{c}}_{s}\left(t\right)$	Context states of device *k* and edge server *s*
$\psi_{k},{\tilde{\psi }}_{s}$	Estimated computing capability under the observed context

**Table 3:** Experimental data setup.

Parameter	Value
*K*	25
*S*	5
$A_{s}^{\max}$	5
$\tau^{\max}$	5 s
Training samples per device	500
Types of labels per device	2
$f_{k}^{\min}\left(c\left(t\right)\right),f_{k}^{\max}\left(c\left(t\right)\right)$	$1\times 10^{9},5\times 10^{9}$
${\tilde{f}}_{s}^{\min}\left(c\left(t\right)\right),{\tilde{f}}_{s}^{\max}\left(c\left(t\right)\right)$	$1\times 10^{11},5\times 10^{11}$
κ	50
*T*	1000

**Table 4:** Estimated single-round training latency under different model splitting schemes.

$I^{1}$, $I^{2}$	2	3	4	5	6	7
1	3.451	3.407	3.275	3.179	2.559	2.338
2	-	6.930	6.815	6.747	6.160	5.897
3	-	-	6.913	6.885	6.284	5.995
4	-	-	-	6.946	6.292	6.078
5	-	-	-	-	6.398	6.133
6	-	-	-	-	-	6.739

## Data Availability

No new data were created or analyzed in this study.

## Appendix A. Proof of Theorem 1

According to assumption 1, based on the second-order Taylor expansion of ${\tilde{F}}_{s}\left({\tilde{w}}_{s}\left(t+1\right)\right)$, we can obtain

(A1) $$\begin{array}{cc} \; & {\tilde{F}}_{s}\left({\tilde{w}}_{s}\left(t+1\right)\right)={\tilde{F}}_{s}\left({\tilde{w}}_{s}\left(t\right)\right)+{\left({\tilde{w}}_{s}\left(t+1\right)-{\tilde{w}}_{s}\left(t\right)\right)}^{T}\nabla {\tilde{F}}_{s}\left({\tilde{w}}_{s}\left(t\right)\right)\\ & +\frac{1}{2}{\left({\tilde{w}}_{s}\left(t+1\right)-{\tilde{w}}_{s}\left(t\right)\right)}^{T}\nabla^{2}{\tilde{F}}_{s}\left({\tilde{w}}_{s}\left(t\right)\right)\left({\tilde{w}}_{s}\left(t+1\right)-{\tilde{w}}_{s}\left(t\right)\right)\\ & \overset{\left(a\right)}{\leq }{\tilde{F}}_{s}\left({\tilde{w}}_{s}\left(t\right)\right)-\frac{1}{2L}\nabla^{2}{\tilde{F}}_{s}\left({\tilde{w}}_{s}\left(t\right)\right)+\frac{1}{2L}∥\nabla {\tilde{F}}_{s}\left({\tilde{w}}_{s}\left(t\right)\right)\\ & -\frac{\sum_{k\in V_{s}\left(t\right)}D_{k}w_{k}\left(t\right)+\sum_{s^{\prime }\in C_{s}\left(t\right)}\sum_{k^{\prime }\in V_{s^{\prime }}\left(t\right)}D_{k^{\prime }}w_{k^{\prime }}\left(t\right)}{\sum_{k\in V_{s}\left(t\right)}D_{k}+\sum_{s^{\prime }\in C_{s}\left(t\right)}\sum_{k^{\prime }\in V_{s^{\prime }}\left(t\right)}D_{k^{\prime }}}{∥}^{2}\\ & \overset{\left(b\right)}{\leq }{\tilde{F}}_{s}\left({\tilde{w}}_{s}\left(t\right)\right)-\frac{1}{2L}\nabla^{2}{\tilde{F}}_{s}\left({\tilde{w}}_{s}\left(t\right)\right)+\frac{4\xi_{2}^{2}\left(\sum_{k\in K}D_{k}-\sum_{k\in V_{s}\left(t\right)}a_{k,s}\left(t\right)D_{k}\right)}{\sum_{k\in K}D_{k}}, \end{array}$$

where steps (a) and (b) are derived form (26) and (29) in [25].

Moreover, according to Assumptions 2 and 3, we further have [43]

(A2) $$\begin{array}{c} ∥\nabla {\tilde{F}}_{s}\left({\tilde{w}}_{s}\left(t\right)\right){∥}^{2}\geq 2\mu \left({\tilde{F}}_{s}\left({\tilde{w}}_{s}\left(t\right)\right)-{\tilde{F}}_{s}\left(w^{*}\right)\right). \end{array}$$

Then, substituting (A2) into (A1), we can obtain

(A3) $$\begin{array}{cc} \; & {\tilde{F}}_{s}\left({\tilde{w}}_{s}\left(t+1\right)\right)-{\tilde{F}}_{s}\left({\bar{w}}_{s}^{*}\right)\leq \left(1-\frac{\mu }{L}\right)\left({\tilde{F}}_{s}\left({\tilde{w}}_{s}\left(t\right)\right)-{\tilde{F}}_{s}\left(w^{*}\right)\right)\\ & +\frac{4\xi_{2}^{2}\left(\sum_{k\in K}D_{k}-\sum_{k\in V_{s}\left(t\right)}a_{k,s}\left(t\right)D_{k}\right)}{\sum_{k\in K}D_{k}}. \end{array}$$

We can derive that

(A4) $$\begin{array}{cc} \xi_{2}^{2}\left(\sum_{k\in K}D_{k}-NU\left(t\right)\right) & =\sum_{k\in V_{s}\left(t\right)}\left(1-a_{k,s}\left(t\right)\right)D_{k}\varpi^{2}\\ \; & \;+\sum_{s^{\prime }\in C_{s}\left(t\right)}\sum_{k^{\prime }\in V_{s^{\prime }}\left(t\right)}\left(1-a_{k^{\prime },s^{\prime }}\left(t\right)u_{s,s^{\prime }}^{E}\left(t\right)\right)D_{k^{\prime }}\varpi^{2}. \end{array}$$

This equation represents the residual contribution of devices that fail to participate locally or whose inter-edge model exchange is unreliable. Equivalently, the successful data mass NU(t) reduces the additive error term in (A3). Applying (A3) recursively yields

(A5) $$\begin{array}{c} \Delta_{t+1}\leq {\left(1-\frac{\mu }{L}\right)}^{t+1}\Delta_{0}+\sum_{\ell =0}^{t}{\left(1-\frac{\mu }{L}\right)}^{\ell }\frac{4\xi_{2}^{2}\left(\sum_{k\in K}D_{k}-NU\left(t-\ell \right)\right)}{\sum_{k\in K}D_{k}}, \end{array}$$

where $\Delta_{t}=E\left({\tilde{F}}_{s}\left({\tilde{w}}_{s}\left(t\right)\right)-{\tilde{F}}_{s}\left(w^{*}\right)\right)$. Since $NU\left(t\right)\geq NU^{\min}$, the geometric sum is upper bounded by $\frac{L}{\mu }$, which leads to the bound in Theorem 1. Therefore, a larger successful participation probability $u_{k}^{D}\left(t\right)$ or a more reliable inter-edge exchange probability $u_{s,s^{\prime }}^{E}\left(t\right)$ directly tightens the loss bound.

Then, applying (A3) recursively, we can complete the proof.
